# Fractional Carbon Dioxide Laser in Treatment of Acne Scars

**DOI:** 10.3889/oamjms.2016.004

**Published:** 2015-12-21

**Authors:** Andrej Petrov, Vesna Pljakovska

**Affiliations:** *Acibadem Sistina Clinical Hospital, Skopje, Republic of Macedonia*

**Keywords:** Acne scars, aesthetic dermatology, carbon dioxide laser systems, subjective evaluation of adverse effects by the therapy, scale of satisfaction by the treatment

## Abstract

**BACKGROUND:**

Scars appear as a result of skin damage during the process of the skin healing. There are two types of acne scars, depending on whether there is a loss or accumulation of collagen: atrophic and hypertrophic. In 80-90% it comes to scars with loss of collagen compared to smaller number of hypertrophic scars and keloids.

**AIM:**

The aim of the study was to determine efficiency and safety of fractional carbon dioxide laser in the treatment of acne scars.

**MATERIAL AND METHODS:**

The study was carried out in Acibadem Sistina Clinical Hospital, Skopje at the Department of Dermatovenerology, with a total of 40 patients treated with fractional carbon dioxide laser (Lutronic eCO2). The study included patients with residual acne scars of a different type.

**RESULTS:**

Comedogenic and papular acne in our material were proportionately presented in 50% of cases, while the other half were the more severe clinical forms of acne - pustular inflammatory acne and nodulocystic acne that leave residual lesions in the form of second, third and fourth grade of scars.

**CONCLUSION:**

The experiences of our work confirm the world experiences that the best result with this method is achieved in dotted ice pick or V-shaped acne scars.

## Introduction

Acne is a common disease. Acne has a prevalence of 90% in adolescents and persists to adulthood in approximately 12-14% of cases, with serious psychological and social implications.

Scars appear as a result of skin damage during the process of the skin healing. There are two types of acne scars, depending on whether there is a loss or accumulation of collagen: atrophic and hypertrophic. In 80-90% it comes to scars with loss of collagen compared to smaller number of hypertrophic scars and keloids.

Atrophic scars are classified into ice pick, rolling and boxcar type. Dotted, ice pick represents 65-70% of atrophic scars, rolling type 20-30% and boxcar type 15-25%.

Ice pick scars are narrow (2 mm), dotted and deep scars, with a wide opening and a deep infundibulum in the form of the letter V.

Rolling scars are wider (up to 5mm) and they reach to the subcutaneous adipose tissue. They give a distorted appearance to the skin, in the form of the letter M.

Boxcar scars have clearly visible vertical edges; they are wider than ice pick scars and take U shape with a broad and visible base [1].

### Qualitative scarring gradation is applied according to Goodman and Baron [2]

The first grade consists of macular hyper- and hypopigmentations that are visible. The second grade includes mild atrophy or hypertrophy, but can be concealed with cosmetics. The third grade consists of moderate atrophic or hypertrophic acne that can not easily be concealed with makeup and can be visible at a distance greater than 50 cm. The most severe grade is highly atrophic or hypertrophic acne visible at a distance greater than 50 cm that are not flattened with pressure of the skin around the scars.

**Table 1:** Qualitative scarring grading system (Goodman and Baron) [2, 3]

Qualitative scarring grading system		In 2007 Goodman and Baron suggested global classification of acne scars in four grades
**Grades of post acne scars**	**Level of disease**	**Clinical features**
1	Macular	These scars can be erythematous, hyper- or hypopigmented flat marks. They do not present a problem of skin contours like other scars but of color.
2	Mild	Mild atrophy or hypertrophic scars may not be visible at a distance of 50 cm or greater, and can be covered with makeup or shadow of shaved beard hair in men.
3	Moderate	Moderate atrophic or hypertrophic scars are visible at social distance of 50 cm or greater, and are not easily covered with mascara or shadow, but still can be flattened by manual pressure of the skin.
4	Severe	Severe atrophic or hypertrophic scars are evident at a distance greater than 50 cm and are not easy covered with makeup, and can not be flattened by manual stretching of the skin.

Acne scars have a major impact on the quality of life, making their treatment to be efficient and to start as early as possible [3].

The treatment of acne scars use a variety of therapeutic modalities, such as chemical peelings [4], methods of tissue augmentation [5], dermabrasion and microdermabrasion, as well as other micro-surgical procedures.

### Carbon dioxide laser

The purpose of minimally invasive treatments in aesthetic dermatology is to obtain better effects with as much smaller thermal trauma to the skin as possible, while keeping the epidermis intact. These methods could be laser and non-laser. Lasers used for that purpose could be non-ablative and ablative. Fractional carbon dioxide laser is the most used today of all laser treatments.

Carbon dioxide laser systems that emit light of 10600 nm maintain the leading position in dermatology. They are the gold standard in the treatment of acne scars. The advantages of carbon dioxide lasers over conventional surgery are less tissue damage and less edema, as well as shorter recovery time. However, the noticed thermal trauma to the surrounding tissue constituted a major problem. [6] The first continuous wave lasers were limited in their use due to their non-specific thermal effect. Improving laser technology led to the use of pulse and ultrapulse laser systems that minimize thermal trauma [7].

eCO_2_ laser is the latest generation of carbon dioxide fractional lasers, with combined fractional technology and deep ablative effect of CO_2_ laser. Micro-ablative columns of laser penetrate deep into the skin with a maximum depth of 2.5 mm. The wavelength of 10600 nm has a high rate of water absorption. Thus greater epidermal damage is avoided, while lateral thermal damage is reduced. In comparison to similar CO_2_ laser systems it causes less damage and also affect the remodelling of collagen fibers in the reticular dermis.

CO_2_ lasers have a double effect – they encourage renewable processes of the wound and incite increased production of myofibroblasts and matrix proteins such as the hyaluronic acid.

Previous clinical and histological studies had shown efficacy of CO_2_ laser skin renewal in atrophic acne cicatrixes, with improvement of 50-80% [8].

Candidates for laser treatment should not receive oral retinoids at least a year, have no active herpes viral infection 6 months prior to the treatment, nor a history of keloids and hypertrophic scars. Patients with a higher skin phenotype are at higher risk of hyperpigmentation than patients with a lower phenotype. All ablative lasers have a risk of complications and adverse effects. The adverse effects of the ablative lasers of first generation are classified as short-lived (bacterial, fungal or herpetic infections) and long-lasting (persistent erythema, hyperpigmentation, scarring) [9, 10].

### Complications

As a complication after laser treatment with carbon dioxide laser, the scars are result of excessive treatment on the area (including excessive energy or density or both of them). It is especially important to take this into account when treating sensitive parts such as eyelids, upper neck, and particularly lower part of the neck, neckline and chest [11, 12]. The new concept of fractional photothermolysis is designed to create microscopic thermal wounds that cause homogeneous thermal damage to a certain level of the skin, in contrast to chemical peeling and traditional laser remodelling. Previous studies have proved the efficiency of fractional photothermolysis in the treatment of acne scars [13] with particular attention to the dark skin to avoid post-inflammatory hyperpigmentations [14]. A new mode of treatment is the so-called fractional photothermolysis, with specific laser devices that create thin microscopic wounds surrounded by undamaged tissue in the patients, which is the advantage of these new devices. These laser systems have a more modest result than traditional laser systems but have very few adverse effects and short recovery period [15].

Topographic analyses showed that the depths of acne scars have significantly improvement that ranges between 43-80%, an average of about 66.8% [16]. Different experiences of numerous authors suggest that combining the technology with fractional photothermolysis is a safe and effective treatment in acne scars. In comparison to the conventional ablation with conventional CO_2_ laser, the adverse effects with fractional photothermolysis are much improved. It is believed that rapid re-epithelialiazation of the surrounding undamaged tissue is responsible for comparatively faster recovery [17-19].

Abnormalities in pigmentation that accompany the laser treatment are always worrying. Alster and West found 36% incidence of hyperpigmentation after using the conventional CO_2_ resurfacing, compared to the ablative photothermolysis (AFR), probably associated with a shorter period of recovery and post-treatment erythema. And treatment strategy to optimize the treatment parameters is to apply an optimal energy and interval between the sessions, and a longer follow-up period.

The aim of the study was to determine efficiency and safety of fractional carbon dioxide laser in the treatment of acne scars.

## Material and Method

The study was carried out in Acibadem Sistina Clinical Hospital, Skopje at the Department of Dermatovenerology, with a total of 40 patients treated with fractional carbon dioxide laser (Lutronic eCO_2_). The study included patients with residual acne scars of a different type.

Exclusionary criteria in the study were the use of oral retinoids in period of 6 months prior to the laser treatment; the use of anticoagulation therapy; age under 17; and the presence of systemic diseases in the patient.

A questionnaire was prepared to collect the data needed for the objectives of the study. Every patient gave consent to participate in the study after being informed about the nature of the treatment and the research.

The treatment was carried out by different protocols depending on the type of lesion and its depth. Patients were treated with eCO_2_ beams of 120 and 300 nm. It used static method directly on the lesion, while the periphery was treated with dynamic module. It used different pulse energy, power, density and number of passes in a single session, and a different number of sessions depending on the clinical features of the patient.

To determine the efficacy and safety of the treatment three methods were used: a questionnaire for subjective evaluation of adverse effects by the therapy (pain, redness, and pigmentation), scale of satisfaction by the treatment and comparison of digital photographs before and after the treatment.

## Results

The average age of patients in our study for men and women collectively was 28.2 years - the average age for men was 28.33, and for women 26.26. The average total duration of acne was 4 years in men and 9 years in women. More severe clinical forms of acne conglobata and nodular cystic acne appeared in men. Comedogenic and papular acne in our material were proportionately presented in 50% of cases, while the other half were the more severe clinical forms of acne - pustular inflammatory acne and nodulocystic acne that leave residual lesions in the form of second, third and fourth grade of scars. Average age of acne appearance was 15.5 years. They started earlier in women than in men. Average duration of scars was 12 years.

**Table 2:** Efflorescence distribution in the population by sex, age and duration

Sex	Men	Women	Total population
		
Average age (range), years	19-38	18-42	18-41
Average age of the patients (range)	28.33	26.26	28.2
Average total duration of acne (range)	4 (1-15) years	9 (1-16) years	6 months-16 years
Average age at appearance of scars (range)	17-38	15-42	15-42
Average total duration of scars (range)	2-16	1-15	1-16

The success of treatment is objectively seen in photographs taken before and after the treatment.

**Figure 1:**
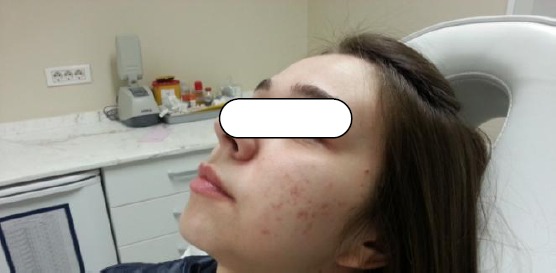
*Before laser treatment*.

**Figure 2:**
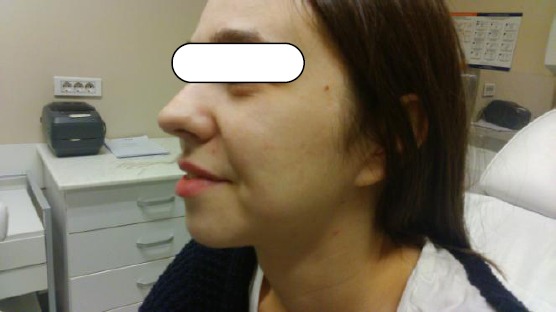
*After 3 treatments with fractional carbon dioxide laser*.

**Table 3:** Preview of scars’ severity before and after the start of treatment and number of laser sessions in average

Type of scars (number of patients treated)	Average score before the treatment (on a scale of scarring grading intensity of 1-5)	Average score after the treatment (on a scale of scarring grading intensity of 1-5)	Average number of treatments
Ice pick (18)	3.46	1.86	3
Rolling (8)	4.1	2.37	4-5
Boxcar (7)	3.5	1.9	3-4
Pigmentations (5)	2.2	1.4	3

## Discussion

Fractional photothermolysis was first described by Anderson and Parrish in 1983 in the journal Science [7].

Laser technology has progressed continuously. The application of fractional carbon dioxide laser in the treatment of acne blemishes was approved by the FDA in 2007.

**Figure 3:**
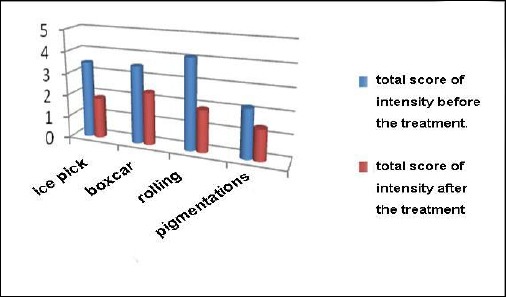
*Scarring intensity on a scale of 1-5 before and after the start of the treatment, according to the scar type classified by Goodman/Baron [2]*.

Numerous researches on the impact of fractional laser on acne scars indicate great success of the treatment [7, 10, 11, 13-15].

Most patients who participated in the researches noticed improvement of the face condition and loss of blemishes [15, 16, 19].

**Figure 4:**
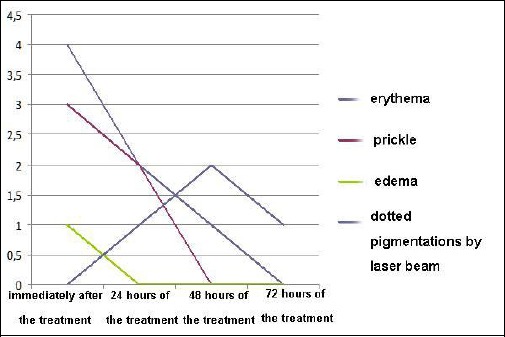
*Transient adverse effects on the skin by laser treatment, on a scale of 1-5/observation based on the average assessment by the doctor, the patient and another doctor at the department*.

According to relevant studies, ablative CO_2_ lasers are ideal for the treatment of grade 3 scars caused by acne [19].

In the treatment of scars, the longer time has passed since the appearance of a certain cicatrix it is more severe and refractory for therapy.

It can be concluded that the selection of patients and response to the therapy in patients participating in this study coincide with world experiences and experiences from great number of research studies.

In conclusion, the results of this study show that fractional laser treatment in these patients is fast and practical. Depending on the scar condition it could be made 6 treatments, and even if the scar is not completely disappeared after the treatment, it significantly improves the look. In 35% of cases there is a complete reduction of scarring, and in 40% a significant reduction of scars with more than 50%. The time period between two treatments is one month. Dotted and atrophic scars showed the best response to treatment with CO_2_ laser. Fractional photothermolysis using fractional eCO_2_ laser is effective and safe method for treating acne scars. The experiences of our work confirm the world experiences that the best result with this method is achieved in dotted ice pick or V-shaped acne scars.
